# Supplementary material for: An Integrative Phylogenetic Analysis of the Genus Rhynchium Spinola (Hymenoptera: Vespidae: Eumeninae) from China Based on Morphology, Genomic Data and Geographical Distribution
Source: Insects. 2025 Feb 16;16(2):217. doi: 10.3390/insects16020217 (PMC11856612; doi:10.3390/insects16020217)
Supplement: Supplementary file 1 [file insects-16-00217-s001.zip › Supplementary_Table_S1- S4.pdf]

Table S1. Sampling information for 35 newly sequenced samples.

Sample ID	Sequencing ID	Accession Number	Location	City	Province	Longitude (E)	Latitude (N)	Altitude (m)	Collection time
CQ	CQ5	SAMN46120559	Jiulongpo District	Chongqing	Chongqing	106.37	29.46	240	2023/9/4
FJ	FJ1	SAMN46120560	Wuyishan City	Nanping	Fujian	117.99	27.83	224	2014/6/25
GD	GD3	SAMN46120561	Chebaling Nature Reserve	Shaoguan	Guangdong	114.26	24.73	362	2002/7/27
GS_1	GS2	SAMN46120562	Zhouqu County	Gannan Tibetan Autonomous Prefecture	Gansu	104.25	33.79	1389	2023/8/17
GS_2	GS3	SAMN46120563	Zhouqu County	Gannan Tibetan Autonomous Prefecture	Gansu	104.25	33.79	1389	2023/8/17
GS_3	GS4	SAMN46120564	Zhouqu County	Gannan Tibetan Autonomous Prefecture	Gansu	104.25	33.79	1389	2023/8/17
GS_4	GS5	SAMN46120565	Zhouqu County	Gannan Tibetan Autonomous Prefecture	Gansu	104.25	33.79	1389	2023/8/17
HEN_1	HE1	SAMN46120566	Taihang Mountains Area	Jiyuan	Henan	112.60	35.11	401	2023/6/15
HEN_2	HE3	SAMN46120567	Taihang Mountains Area	Jiyuan	Henan	112.60	35.11	401	2023/6/15
HEN_3	HE4	SAMN46120568	Taihang Mountains Area	Jiyuan	Henan	112.60	35.11	401	2023/6/15
HNBT	HN5	SAMN46120569	Shiqun Village	Baoting Li and Miao Autonomous County	Hainan	109.63	18.57	85	2021/5/30
HNBWL	HN6	SAMN46120570	Bawangling	Changjiang Li Autonomous County	Hainan	109.09	19.12	451	2020/6/21

HNTGL	HN7	SAMN46120571	Tongguling Scenic Area	Wenchang	Hainan	111.03	19.65	300	2024/4/11
HNBS	HN8	SAMN46120572	Bangxi Reserve	Baisha Li Autonomous County	Hainan	109.09	19.39	49	2021/4/27
HUN	HUN1	SAMN46120573	Daba Town	Zhangjiajie	Hunan	110.17	29.54	568	2016/7/28
YNKM1_1	R1	SAMN46120574	Dianchi Road	Kunming	Yunnan	102.68	24.98	1886	2015/6/4
SXLL_1	R10	SAMN46120575	Jiaokou County	Luliang	Shanxi	111.32	36.92	1127	2019/7/31
GZ1	R11	SAMN46120576	Jiangkou County	Tongren	Guizhou	108.70	27.66	434	2015/6/29
GZ2	R12	SAMN46120577	Songtao County	Tongren	Guizhou	108.99	28.09	441	2018/8/2
SXLF1	R14	SAMN46120578	Xi County	Linfen	Shanxi	111.01	36.74	1111	2019/8/1
YNKM1_2	R2	SAMN46120579	Dianchi Road	Kunming	Yunnan	102.68	24.98	1886	2015/6/4
YNKM1_3	R3	SAMN46120580	Dianchi Road	Kunming	Yunnan	102.68	24.98	1886	2015/6/4
YNKM1_4	R4	SAMN46120581	Dianchi Road	Kunming	Yunnan	102.68	24.98	1886	2015/6/4
YNHH1_1	R5	SAMN46120582	Hekou County	Honghe Hani and Yi Autonomous Prefecture	Yunnan	103.96	22.63	286	2015/7/18
YNHH1_2	R6	SAMN46120583	Hekou County	Honghe Hani and Yi Autonomous Prefecture	Yunnan	103.96	22.63	286	2015/7/18
YNHH1_3	R7	SAMN46120584	Hekou County	Honghe Hani and Yi Autonomous Prefecture	Yunnan	103.96	22.63	286	2015/7/18
YNHH1_4	R8	SAMN46120585	Hekou County	Honghe Hani and Yi Autonomous Prefecture	Yunnan	103.96	22.63	286	2015/7/18
SXLL_2	R9	SAMN46120586	Jiaokou	Luliang	Shanxi	111.32	36.92	1127	2019/7/31
SXLF2	SX4	SAMN46120587	Fenxi County	Linfen	Shanxi	111.54	36.66	986	2023/7/22
YNDH	YN10	SAMN46120588	Yingjiang County	Dehong	Yunnan	97.59	24.67	674	2017/8/19
YNBN1	YN11	SAMN46120589	Gasa Town	Xishuangbanna Dai Autonomous Prefecture	Yunnan	100.68	22.13	717	2017/8/3

YNBN2	YN12	SAMN46120590	Mengla County	Xishuangbanna Dai Autonomous Prefecture	Yunnan	101.72	21.25	762	2015/7/28
YNKM2	YN5	SAMN46120591	Chongming County	Kunming	Yunnan	103.02	25.24	1929	2014/7/13
YNPE1	YN6	SAMN46120592	Zhenyuan County	Pu'er	Yunnan	100.91	24.04	1136	2017/6/9
YNHH2	YN7	SAMN46120593	Luchun County	Honghe Hani and Yi Autonomous Prefecture	Yunnan	102.16	23.04	1273	2022/8/4

Table S2. Data Information Downloaded from NCBI.

Sample ID	Accession Number	Seqtype	Location	City	Province	Collection time	Sample source
YNBS	SAMN36845277	whole genomic	Longyang District	Baoshan	Yunnan	2017/8/12	Dai et al, 2023
GX	SAMN36845333	whole genomic	Jinxiu County	Laibin	Guangxi	2016/5/29	
Outgroups1	SAMN36845335	whole genomic	Zhenyuan County	Pu'er	Yunnan	2017/6/9	
Outgroups2	SAMN36845336	whole genomic	Yangshuo County	Guilin	Guangxi	2015/7/27	
YNPE2	MK051030	Mitogenome	Zhenyuan County	Pu'er	Yunnan	2017/6/9	Luo et al, 2022
YNBN3	MK051031	Mitogenome	Wild Elephant Valley	Xishuangbanna Dai Autonomous Prefecture	Yunnan	2017/8/3	
YNBN4	MK051032	Mitogenome	Mengla County	Xishuangbanna Dai Autonomous Prefecture	Yunnan	2017/8/5	
Outgroups3	MK051021	Mitogenome	Yangshuo County	Guilin	Guangxi	2015/7/27	
Outgroups4	MK051022	Mitogenome	Mengla County	Xishuangbanna Dai Autonomous Prefecture	Yunnan	2017/8/5	

Table S3. Genome assembly information for 37 samples of the genus *Rhynchium*.

Sample ID	Average Read Coverage (X)	BUSCO Completeness (%)	The Number of Scaffolds	Assembly Size (Mb)	Max Read Length (kb)	N50 Scaffold (kb)	GC (%)
YNBS	78.85	94.60%	42,348	194.79	1170.58	134.32	37.24
GX	78.26	94.00%	48,619	196.26	1281.60	105.73	37.24
CQ	33.05	89.00%	24,500	185.91	292.75	22.99	37.51
FJ	26.44	89.20%	306,444	232.39	363.08	15.20	36.87
GD	29.66	77.20%	241,082	207.17	133.52	6.54	37.96
GS_1	29.44	75.50%	280,055	208.71	169.17	5.83	37.91
GS_2	38.91	39.00%	73,423	157.91	97.76	4.22	39.01
GS_3	28.22	87.10%	247,057	217.71	195.46	17.33	37.27
GS_4	28.92	83.50%	242,821	212.48	240.56	11.19	37.68
HEN_1	31.00	64.00%	79,336	198.19	959.97	4.36	39.67
HEN_2	33.04	87.30%	28,186	185.96	287.49	19.38	37.45
HEN_3	31.93	77.70%	46,484	192.45	779.23	9.20	38.72
HNBT	33.25	87.20%	29,505	184.76	206.34	19.94	37.59
HNBWL	31.00	92.30%	56,961	198.19	550.05	41.19	37.00
HNTGL	31.34	86.80%	97,969	196.03	326.72	17.11	37.65
HNBS	30.26	90.90%	86,300	203.02	351.57	28.77	36.90
HUN	28.66	89.70%	216,339	214.38	242.11	23.62	37.25
YNKM1_1	30.77	89.30%	103,148	199.66	339.92	25.07	37.48
SXLL_1	28.84	88.10%	213,519	213.05	263.05	20.28	37.41
GZ1	31.09	90.30%	85,184	197.65	427.05	30.42	37.42
GZ2	31.62	77.80%	103,275	194.30	397.24	12.54	37.70

SXLF1	31.56	89.70%	79,198	194.70	484.22	23.71	37.62
YNKM1_2	32.39	83.60%	106,505	189.71	129.66	7.82	38.57
YNKM1_3	30.87	89.10%	98,903	199.04	288.41	22.94	37.41
YNKM1_4	27.64	88.30%	239,923	222.30	310.69	18.60	37.55
YNHH1_1	31.03	91.40%	83,330	198.03	519.72	37.84	37.40
YNHH1_2	30.92	89.30%	95,009	198.68	387.80	25.70	37.42
YNHH1_3	31.08	91.10%	84,279	197.68	528.03	35.06	37.41
YNHH1_4	31.12	89.70%	87,402	197.41	352.68	27.83	37.44
SXLL_2	31.17	90.40%	84,282	197.09	453.01	30.97	37.45
SXLF2	45.66	33.60%	84,897	134.55	158.22	2.53	41.16
YNDH	30.98	88.80%	95,195	198.29	336.45	25.46	37.44
YNBN1	30.72	90.00%	97,297	200.01	378.12	28.75	37.32
YNBN2	28.71	87.40%	214,234	214.03	263.28	19.20	37.27
YNKM2	27.69	85.90%	212,845	221.91	210.90	14.56	38.12
YNPE1	30.90	91.20%	84,720	198.82	720.62	36.98	37.33
YNHH2	31.23	92.10%	65,365	196.72	637.98	44.82	37.36

Table S4. Intraspecific pairwise genetic distances base on *COI.*

	HNBWL	HNBS	YNKM1_1	YNKM1_2	YNKM1_3	YNKM1_4	YNKM2	YNPE1	YNHH2	YNPE2	GD	FJ	GX	GS_3	HEN_1	HNBT	HNTGL	HUN	SXLL_1	YNHH1_3
HNBWL																				
HNBS	0.0000																			
YNKM1_1	0.1392	0.1392																		
YNKM1_2	0.1400	0.1400	0.0007																	
YNKM1_3	0.1400	0.1400	0.0007	0.0000																
YNKM1_4	0.1400	0.1400	0.0007	0.0000	0.0000															
YNKM2	0.1400	0.1400	0.0007	0.0000	0.0000	0.0000														
YNPE1	0.1400	0.1400	0.0007	0.0000	0.0000	0.0000	0.0000													
YNHH2	0.1400	0.1400	0.0013	0.0007	0.0007	0.0007	0.0007	0.0007												
YNPE2	0.1403	0.1403	0.0007	0.0000	0.0000	0.0000	0.0000	0.0000	0.0007											
GD	0.1260	0.1260	0.0730	0.0737	0.0737	0.0737	0.0737	0.0737	0.0730	0.0739										
FJ	0.1268	0.1268	0.0737	0.0745	0.0745	0.0745	0.0745	0.0745	0.0737	0.0746	0.0006									
GX	0.1268	0.1268	0.0737	0.0745	0.0745	0.0745	0.0745	0.0745	0.0737	0.0746	0.0006	0.0000								
GS_3	0.1260	0.1260	0.0723	0.0730	0.0730	0.0730	0.0730	0.0730	0.0737	0.0731	0.0032	0.0026	0.0026							
HEN_1	0.1268	0.1268	0.0730	0.0737	0.0737	0.0737	0.0737	0.0737	0.0745	0.0739	0.0026	0.0019	0.0019	0.0006						
HNBT	0.1268	0.1268	0.0737	0.0745	0.0745	0.0745	0.0745	0.0745	0.0737	0.0746	0.0006	0.0000	0.0000	0.0026	0.0019					
HNTGL	0.1268	0.1268	0.0737	0.0745	0.0745	0.0745	0.0745	0.0745	0.0737	0.0746	0.0006	0.0000	0.0000	0.0026	0.0019	0.0000				
HUN	0.1268	0.1268	0.0730	0.0737	0.0737	0.0737	0.0737	0.0737	0.0745	0.0739	0.0026	0.0019	0.0019	0.0006	0.0000	0.0019	0.0019			
SXLL_1	0.1268	0.1268	0.0730	0.0737	0.0737	0.0737	0.0737	0.0737	0.0745	0.0739	0.0026	0.0019	0.0019	0.0006	0.0000	0.0019	0.0019	0.0000		
YNHH1_3	0.1268	0.1268	0.0737	0.0745	0.0745	0.0745	0.0745	0.0745	0.0737	0.0746	0.0006	0.0000	0.0000	0.0026	0.0019	0.0000	0.0000	0.0019	0.0019	
SXLF2	0.1268	0.1268	0.0730	0.0737	0.0737	0.0737	0.0737	0.0737	0.0745	0.0739	0.0026	0.0019	0.0019	0.0006	0.0000	0.0019	0.0019	0.0000	0.0000	0.0019
YNDH	0.1268	0.1268	0.0737	0.0745	0.0745	0.0745	0.0745	0.0745	0.0737	0.0746	0.0006	0.0000	0.0000	0.0026	0.0019	0.0000	0.0000	0.0019	0.0019	0.0000
YNBN2	0.1268	0.1268	0.0737	0.0745	0.0745	0.0745	0.0745	0.0745	0.0737	0.0746	0.0006	0.0000	0.0000	0.0026	0.0019	0.0000	0.0000	0.0019	0.0019	0.0000
YNBN3	0.1268	0.1268	0.0739	0.0746	0.0746	0.0746	0.0746	0.0746	0.0739	0.0746	0.0007	0.0000	0.0000	0.0026	0.0020	0.0000	0.0000	0.0020	0.0020	0.0000
YNBN4	0.1268	0.1268	0.0739	0.0746	0.0746	0.0746	0.0746	0.0746	0.0739	0.0746	0.0007	0.0000	0.0000	0.0026	0.0020	0.0000	0.0000	0.0020	0.0020	0.0000
SXLF1	0.1292	0.1292	0.0737	0.0745	0.0745	0.0745	0.0745	0.0745	0.0752	0.0746	0.0124	0.0118	0.0118	0.0118	0.0111	0.0118	0.0118	0.0111	0.0111	0.0118
CQ	0.1292	0.1292	0.0737	0.0745	0.0745	0.0745	0.0745	0.0745	0.0752	0.0746	0.0124	0.0118	0.0118	0.0118	0.0111	0.0118	0.0118	0.0111	0.0111	0.0118
YNBS	0.1284	0.1284	0.0730	0.0737	0.0737	0.0737	0.0737	0.0737	0.0745	0.0739	0.0131	0.0124	0.0124	0.0124	0.0118	0.0124	0.0124	0.0118	0.0118	0.0124
GS_1	0.1300	0.1300	0.0745	0.0752	0.0752	0.0752	0.0752	0.0752	0.0760	0.0754	0.0131	0.0124	0.0124	0.0124	0.0118	0.0124	0.0124	0.0118	0.0118	0.0124
GS_2	0.1268	0.1268	0.0715	0.0723	0.0723	0.0723	0.0723	0.0723	0.0730	0.0724	0.0131	0.0137	0.0137	0.0137	0.0131	0.0137	0.0137	0.0131	0.0131	0.0137
GS_4	0.1276	0.1276	0.0723	0.0730	0.0730	0.0730	0.0730	0.0730	0.0737	0.0731	0.0137	0.0131	0.0131	0.0131	0.0124	0.0131	0.0131	0.0124	0.0124	0.0131
HEN_2	0.1292	0.1292	0.0737	0.0745	0.0745	0.0745	0.0745	0.0745	0.0752	0.0746	0.0137	0.0131	0.0131	0.0131	0.0124	0.0131	0.0131	0.0124	0.0124	0.0131
HEN_3	0.1292	0.1292	0.0737	0.0745	0.0745	0.0745	0.0745	0.0745	0.0752	0.0746	0.0137	0.0131	0.0131	0.0131	0.0124	0.0131	0.0131	0.0124	0.0124	0.0131
GZ1	0.1292	0.1292	0.0737	0.0745	0.0745	0.0745	0.0745	0.0745	0.0752	0.0746	0.0124	0.0118	0.0118	0.0118	0.0111	0.0118	0.0118	0.0111	0.0111	0.0118
GZ2	0.1292	0.1292	0.0737	0.0745	0.0745	0.0745	0.0745	0.0745	0.0752	0.0746	0.0124	0.0118	0.0118	0.0118	0.0111	0.0118	0.0118	0.0111	0.0111	0.0118
YNHH1_1	0.1284	0.1284	0.0730	0.0737	0.0737	0.0737	0.0737	0.0737	0.0745	0.0739	0.0131	0.0124	0.0124	0.0124	0.0118	0.0124	0.0124	0.0118	0.0118	0.0124
YNHH1_2	0.1284	0.1284	0.0730	0.0737	0.0737	0.0737	0.0737	0.0737	0.0745	0.0739	0.0131	0.0124	0.0124	0.0124	0.0118	0.0124	0.0124	0.0118	0.0118	0.0124
YNHH1_4	0.1284	0.1284	0.0730	0.0737	0.0737	0.0737	0.0737	0.0737	0.0745	0.0739	0.0131	0.0124	0.0124	0.0124	0.0118	0.0124	0.0124	0.0118	0.0118	0.0124
SXLL_2	0.1292	0.1292	0.0737	0.0745	0.0745	0.0745	0.0745	0.0745	0.0752	0.0746	0.0124	0.0118	0.0118	0.0118	0.0111	0.0118	0.0118	0.0111	0.0111	0.0118
YNBN1	0.1292	0.1292	0.0737	0.0745	0.0745	0.0745	0.0745	0.0745	0.0752	0.0746	0.0137	0.0131	0.0131	0.0131	0.0124	0.0131	0.0131	0.0124	0.0124	0.0131

	SXLF2	YNDH	YNBN2	YNBN3	YNBN4	SXLF1	CQ	YNBS	GS_1	GS_2	GS_4	HEN_2	HEN_3	GZ1	GZ2	YNHH1_1	YNHH1_2	YNHH1_4	SXLL_2	YNBN1
HNBWL																				
HNBS																				
YNKM1_1																				
YNKM1_2																				
YNKM1_3																				
YNKM1_4																				
YNKM2																				
YNPE1																				
YNHH2																				
YNPE2																				
GD																				
FJ																				
GX																				
GS_3																				
HEN_1																				
HNBT																				
HNTGL																				
HUN																				
SXLL_1																				
YNHH1_3																				
SXLF2																				
YNDH	0.0019																			
YNBN2	0.0019	0.0000																		
YNBN3	0.0020	0.0000	0.0000																	
YNBN4	0.0020	0.0000	0.0000	0.0000																
SXLF1	0.0111	0.0118	0.0118	0.0118	0.0118															
CQ	0.0111	0.0118	0.0118	0.0118	0.0118	0.0000														
YNBS	0.0118	0.0124	0.0124	0.0125	0.0125	0.0006	0.0006													
GS_1	0.0118	0.0124	0.0124	0.0125	0.0125	0.0006	0.0006	0.0013												
GS_2	0.0131	0.0137	0.0137	0.0138	0.0138	0.0019	0.0019	0.0013	0.0026											
GS_4	0.0124	0.0131	0.0131	0.0132	0.0132	0.0013	0.0013	0.0006	0.0019	0.0006										
HEN_2	0.0124	0.0131	0.0131	0.0132	0.0132	0.0013	0.0013	0.0006	0.0019	0.0019	0.0013									
HEN_3	0.0124	0.0131	0.0131	0.0132	0.0132	0.0013	0.0013	0.0006	0.0019	0.0019	0.0013	0.0000								
GZ1	0.0111	0.0118	0.0118	0.0118	0.0118	0.0000	0.0000	0.0006	0.0006	0.0019	0.0013	0.0013	0.0013							
GZ2	0.0111	0.0118	0.0118	0.0118	0.0118	0.0000	0.0000	0.0006	0.0006	0.0019	0.0013	0.0013	0.0013	0.0000						
YNHH1_1	0.0118	0.0124	0.0124	0.0125	0.0125	0.0006	0.0006	0.0000	0.0013	0.0013	0.0006	0.0006	0.0006	0.0006	0.0006					
YNHH1_2	0.0118	0.0124	0.0124	0.0125	0.0125	0.0006	0.0006	0.0000	0.0013	0.0013	0.0006	0.0006	0.0006	0.0006	0.0006	0.0000				
YNHH1_4	0.0118	0.0124	0.0124	0.0125	0.0125	0.0006	0.0006	0.0000	0.0013	0.0013	0.0006	0.0006	0.0006	0.0006	0.0006	0.0000	0.0000			
SXLL_2	0.0111	0.0118	0.0118	0.0118	0.0118	0.0000	0.0000	0.0006	0.0006	0.0019	0.0013	0.0013	0.0013	0.0000	0.0000	0.0006	0.0006	0.0006		
YNBN1	0.0124	0.0131	0.0131	0.0132	0.0132	0.0013	0.0013	0.0006	0.0019	0.0019	0.0013	0.0013	0.0013	0.0013	0.0013	0.0006	0.0006	0.0006	0.0013	
